# Fullerene-Structured MoSe_2_ Hollow Spheres Anchored on Highly Nitrogen-Doped Graphene as a Conductive Catalyst for Photovoltaic Applications

**DOI:** 10.1038/srep13214

**Published:** 2015-08-17

**Authors:** Enbing Bi, Han Chen, Xudong Yang, Fei Ye, Maoshu Yin, Liyuan Han

**Affiliations:** 1 State Key Laboratory of Metal Matrix Composites, School of Materials Science and Engineering, Shanghai Jiao Tong University, Shanghai 200240, China; 2 Photovoltaic Materials Unit, National Institute for Materials Science, Tsukuba, Ibaraki 305-0047, Japan

## Abstract

A conductive catalyst composed of fullerene-structured MoSe_2_ hollow spheres and highly nitrogen-doped graphene (HNG-MoSe_2_) was successfully synthesized via a wet chemical process. The small molecule diethylenetriamine, which was used during the process, served as a surfactant to stabilize the fullerene-structured MoSe_2_ hollow spheres and to provide a high content of nitrogen heteroatoms for graphene doping (ca. 12% N). The superior synergistic effect between the highly nitrogen-doped graphene and the high surface-to-volume ratio MoSe_2_ hollow spheres afforded the HNG-MoSe_2_ composite high conductivity and excellent catalytic activity as demonstrated by cyclic voltammetry, electrochemical impedance spectroscopy and Tafel measurements. A dye-sensitized solar cell (DSSC) prepared with HNG-MoSe_2_ as a counter electrode exhibited a conversion efficiency of 10.01%, which was close to that of a DSSC with a Pt counter electrode (10.55%). The synergy between the composite materials and the resulting highly efficient catalysis provide benchmarks for preparing well-defined, graphene-based conductive catalysts for clean and sustainable energy production.

Dye-sensitized solar cells (DSSCs) have received considerable attention because of their low cost, easy fabrication, and high power conversion efficiency^1^,^2^. A standard DSSC consists of a transparent conducting oxide, a TiO_2_ photoanode coated with dye, an electrolyte, and a counter electrode (CE). The key role of the CE is to transfer electrons from the external circuit to the electrolyte and to catalyze the reduction of the redox couple^3^,^4^,^5^. Therefore, the main requirements for an effective CE include (1) high conductivity for charge transfer and (2) efficient catalytic activity for regeneration of the redox couple^6^. Platinum-based materials are commonly used as CEs, but their high cost, low abundance, and sensitivity to electrolytes hinder their large-scale utilization in DSSCs. Thus, developing a low-cost, highly conductive catalyst for reduced redox electrolyte still remains a priority.

Recently, nanostructured MoX_2_ (X: S, Se) materials synthesized via physical/chemical procedures have received attention owing to their potential for utilization in electronic sensors, hydrogen evolution schemes, lithium ion batteries, supercapacitors, and DSSCs^7^,^8^,^9^,^10^,^11^. Generally, MoX_2_ tends to have a layered structure, analogous to that of graphene, that gives the material versatile and tunable photoelectrochemical properties. However, the widespread commercialization of these innovative nanostructured MoX_2_ materials, particularly as conductive catalysts, has been hindered by the unsatisfactory catalytic activity and poor conductivity that are observed when the layers are stacked in the order X-Mo-X^12^,^13^. To address these problems, researchers initially proposed engineering the surface structure of MoS_2_ nanosheets to preferentially expose active edge sites to enhance electrocatalysis^14^. A rapid sulfurization/selenization process was further developed to obtain highly ordered MoSe_2_ nanosheets on curved and rough surfaces to increase the number of active edges^15^. Xie *et al.* induced defects in MoS_2_ ultrathin nanosheets to improve catalytic activity^16^. Moreover, few-layer MoSe_2_ selenizationon Mo metal has been shown to decrease the sheet resistance of a CEused in DSSCs^13^. Recently, hybridization of MoX_2_ with conductive graphene has shown promise to boost the conductivity and catalytic activity of the material at same time^17^,^18^. However, most studies to date have been focused on the design of 2D MoX_2_ nanosheets on graphene: the growth of such 2D nanosheets on graphene surfaces is facilitated by the highly matched structures of the two materials (i.e., both are sheet-like). In contrast, the synthesis of MoX_2_ structures other than sheets on graphene can be more challenging. Despite this challenge, from the viewpoint of advancing in-depth scientific research and cost-effective industrial-scale production, it is worthwhile to realize new structures and properties of MoX_2_-based materials. One such structure, the hollow sphere, is now playing an important role in energy conversion and storage technologies. The unique structure of hollow spheres provides an enhanced surface-to-volume ratio and reduced charge transport lengths. Hollow spheres have been used in numerous assemblies including as CEs in solar cells and as high-performance electrodes in lithium ion batteries and supercapacitors^19^,^20^,^21^,^22^. Therefore, it is desirable to synthesize hollow spheres of MoX_2_ (X: S, Se), particularly by means of solution-based processes that do not require hard templates, to observe their new properties and applications. Additionally, functionalized graphene, such as nitrogen-doped graphene, is very effective for charge transport^23^,^24^. Recently, we synthesized a novel core-shell catalyst composed of nitrogen-doped graphene shelled on cobalt sulfide nanocrystals, and the resulting DSSC efficiency is 10.7%, which is comparable to that observed for DSSCs with Pt CEs^25^. However, there are also some problems that we found: (1) the conductivity of these nanocrystals was limited due to the low amount of nitrogen (<5%) in the graphene. Nitrogen incorporation in graphene is expected to improve the catalytic activity of the composites since it enhances the electron-donating ability of the graphene; (2) It is difficult to obtain CoS nanocrystals with high pure phase and moreover the CoS nanocrystals is not very stable in the air condition because it apt to form the hydroxide.

Herein, we report, for the first time, the synthesis of a hybrid catalyst composed of fullerene-structured MoSe_2_ hollow spheres anchored on highly nitrogen-doped graphene (referred to herein as HNG) to simultaneously optimize conductivity and catalytic activity. Since the conductivity and catalytic activity of the composites depended on the degree of N-doping in the graphene as well as the MoSe_2_ structure, we highlight the importance of incorporating the small molecule diethylenetriamine (DETA) to provide a high N content for doping graphene and to serve as a surfactant for fullerene-structured MoSe_2_ hollow spheres. More importantly, our HNG-MoSe_2_ hybrid catalyst exhibits excellent catalytic activity and conductivity comparable to that of Pt due to the superior synergy between HNG and MoSe_2_.The resulting DSSC shows a conversion efficiency of 10.01%, which is close to that observed for Pt-based DSSC (10.55%).

## Results

Initially, we selected graphene (G), obtained from treating graphene oxide (GO) with ammonia, as the substrate for MoSe_2_ because GO adsorbs MoSe_2_ too strongly, resulting in the formation of layered MoSe_2_ nanosheets that lie on or stack parallel to the GO surface due to large amount of functional groups of GO^26^,^27^. In addition, the application of graphene oxide in DSSCs is restricted due to its insulating property and solubility caused by the presence of hydrophilic hydroxyl/carboxyl groups and intercalated water molecules^28^. Briefly, G was obtained by treating GO with ammonia (NH_3_·H_2_O), which released NH_3_ gas and converted GO to G with a large surface area for MoSe_2_ nanosheet growth (Fig. S1a,b). The resulting ultrathin G nanosheet had a thickness of 2.583 nm, which was slightly thicker than that of GO as confirmed by AFM and TEM images in (Fig. S1c,d). Next, to form fullerene-structured hollow spheres of MoSe_2_ on the G surface while also creating HNG, we used diethylenetriamine (DETA), which served both as a surfactant and as a N source for creating HNG. For comparison, we also synthesized G-MoSe_2_ in the absence of DETA.

**Figure 1:**
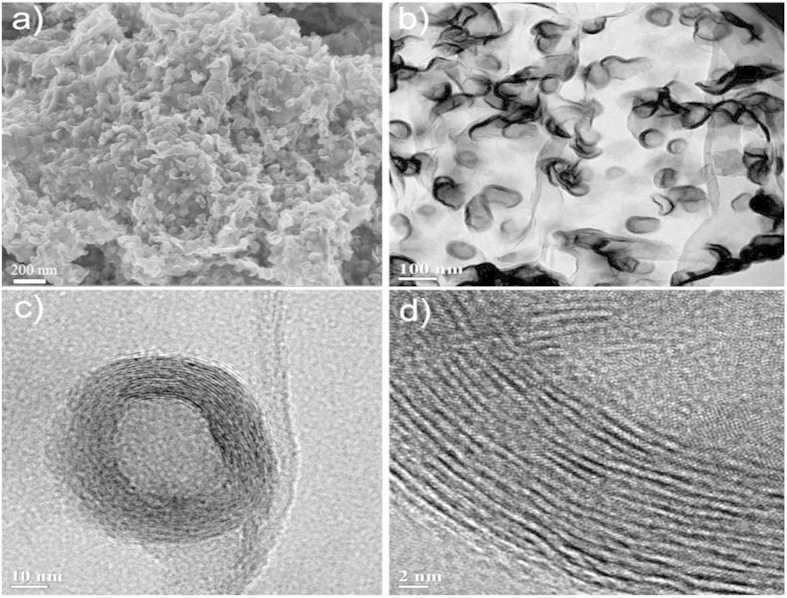
(**a**) SEM image of HNG-MoSe_2_. (**b**) TEM image of HNG-MoSe_2_. (**c,d**) HR-TEM images of MoSe_2_ showing a closed fullerene-like hollow structure on an HNG layer.

We investigated the morphology and composition of HNG-MoSe_2_ and G-MoSe_2_ hybrids by scanning electron microscopy (SEM) and transmission electron microscopy (TEM). As shown in Fig. 1a–c, in the presence of DETA, the fullerene-structured MoSe_2_ hollow spheres were homogeneously distributed on HNG. Closed hollow MoSe_2_ spheres with diameters of 60–100 nm and thicknesses of 8–12 nm were well-dispersed on the surface of ultrathin HNG: the two layered spacing can be identified to be around 7.7 Å; this spacing is wider than that observed for MoSe_2_ synthesized previously without surfactants^27^,^29^. This observed interplanar spacing also indicates that the hollow spheres contained 12–15 layers of MoSe_2_. In addition, the HNG formed interconnected 2D conductive networks related to MoSe_2_; these networks have the potential to enhance the conductivity of the hybrid for efficiently transferring external electrons to MoSe_2_ hollow spheres in a DSSC. At the same time, the unique hollow structure of the MoSe_2_ spheres should provide an enhanced surface-to-volume ratio for increasing the contact area available for electrolyte redox couples and for reducing charge transport lengths, as reported previously^30^. This unique synergistic effect is beneficial for simultaneous optimization of the conductivity and catalytic activity in the HNG-MoSe_2_. The elemental composition of HNG-MoSe_2_ was determined by energy-dispersive X-ray (EDX) analysis (Fig. S2), which indicated that the hybrid contains mostly Mo, Se, C, O, and N. The Mo:Se atomic ratio calculated for all samples was 1:2.03, which is close to the stoichiometric ratio of Mo:Se for MoSe_2_. The amount of MoSe_2_ loading in the HNG-MoSe_2_ hybrid was about 52.4 wt% based on thermal gravimetric measurement (Fig. S3). Moreover, in the absence of DETA, the poorly crystalline MoSe_2_ exhibited a typical ultrathin nanosheet structure on G (G-MoSe_2_, shown in Fig. S5).

**Figure 2:**
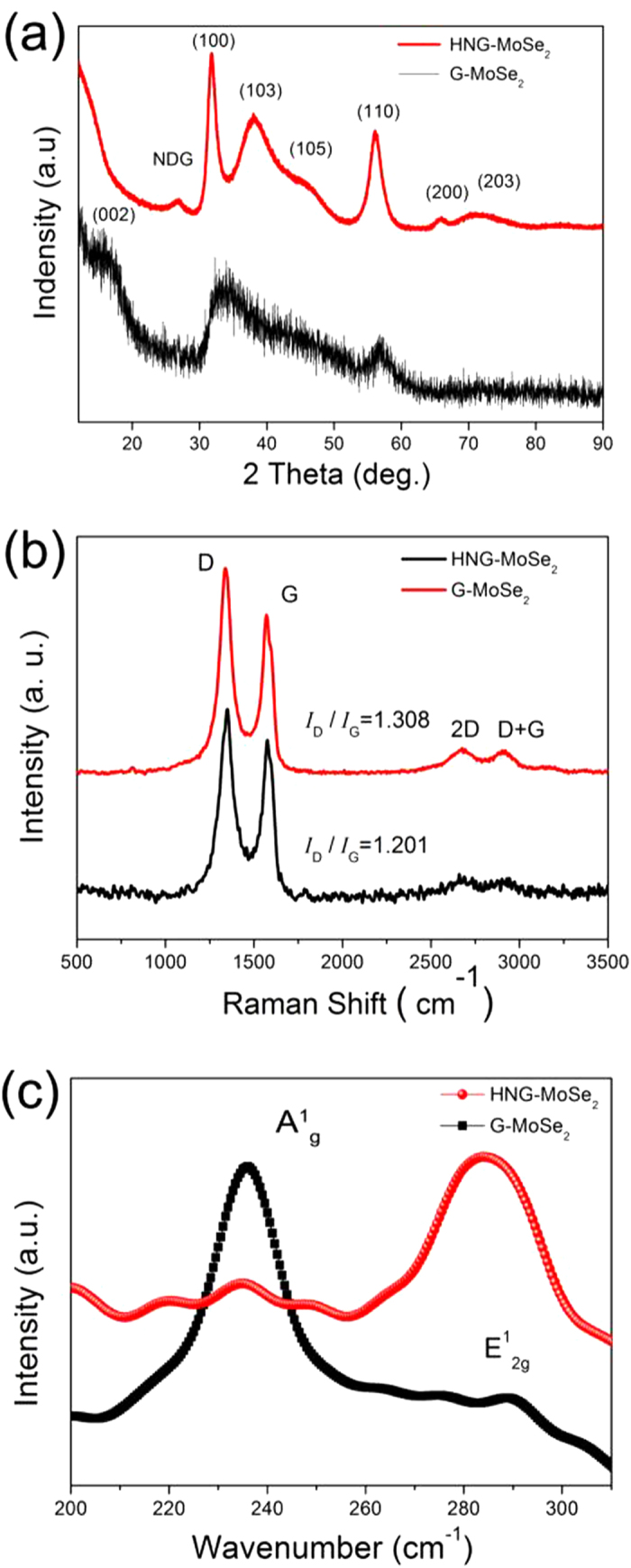
(**a**) XRD spectra of HNG-MoSe_2_ and G-MoSe_2_. (**b,c**) Raman spectra of HNG-MoSe_2_ and G-MoSe_2_ in the high (**b**) and low (**c**) wave number regions.

To gain additional structural information about HNG-MoSe_2_ and G-MoSe_2_, we subjected the samples to X-ray diffraction (XRD) analysis (Fig. 2a). The HNG-MoSe_2_ XRD pattern clearly shows three high-intensity diffraction peaks at 2θ = 13.0°, 33.8°, and 57.1°, corresponding to the (002), (100), and (110) planes of standard hexagonal MoSe_2_ (JCPDS 37-1492) and indicating the high crystallization of the structures corresponding to the selected-area diffraction (SAED) results observed for G-MoSe_2_ (Fig. S4)^27^. In contrast, the weak G-MoSe_2_ XRD pattern indicates that G-MoSe_2_ synthesized without DETA had poor crystallization, in agreement with the SAED results observed for G-MoSe_2_ (Fig. S5c). Therefore, the presence of DETA appeared to be beneficial for the crystallization of MoSe_2_ on HNG. In addition, the weak, broad peak observed at 2θ = 26.1° is ascribed to stacked HNG sheets. This HNG peak might have been partially obscured due to the uniformly distributed MoSe_2_ covering the HNG surface^17^. Raman spectra of HNG-MoSe_2_ and G-MoSe_2_ present a D band around 1343 cm^–1^ (arising from sp^3^ defect sites in graphene), a G band around 1574 cm^–1^ (arising from sp^2^-bonded pairs in graphene), a 2D band around 2681 cm^–1^, and a D + G band around 2910 cm^–1^, which are all characteristic of Raman spectra of graphene (Fig. 2b). The *I*_D_/*I*_G_ ratio in the Raman spectra was used to evaluate the disorder in the graphene^31^. The *I*_D_/*I*_G_ value for HNG-MoSe_2_ was higher than that for G-MoSe_2_, indicating that the HNG had a high degree of defects, most likely because of the high density of N-doping in it. Notably, the Raman peak corresponding to the out-of plane Mo−S phonon mode (A^1^_g_) was preferentially excited for edge-terminated films, whereas the in-plane Mo−S phonon mode (E^1^_2g_) was preferentially excited for terrace-terminated films. The ratio of the relative integrated intensities of these two Raman modes can provide information about the texture of the MoSe_2_ film: an A^1^_g_ mode intensity that is higher than the E^1^_2g_ mode intensity indicates the formation of an edge-terminated structure^32^.

The A^1^_g_ and E^1^_2g_ Raman modes of MoSe_2_ in HNG-MoSe_2_ were observed at 236 and 284 cm^−1^, respectively (Fig. 2c). The A^1^_g_/E^1^_2g_ intensity ratio for MoSe_2_ in G-MoSe_2_ is higher than that for MoSe_2_ in HNG-MoSe_2_ (A^1^_g_:E^1^_2g_ = 8:1 vs A^1^_g_:E^1^_2g_ = 1:8). These A^1^_g_/E^1^_2g_ intensity ratios confirm the presence of exposed edge planes of MoSe_2_ ultrathin nanosheets on G and exposed basal planes of MoSe_2_ fullerene-structured hollow spheres on HNG, respectively. These results are consistent with the SEM and TEM images in Fig. 1 and Fig. S5.

**Figure 3:**
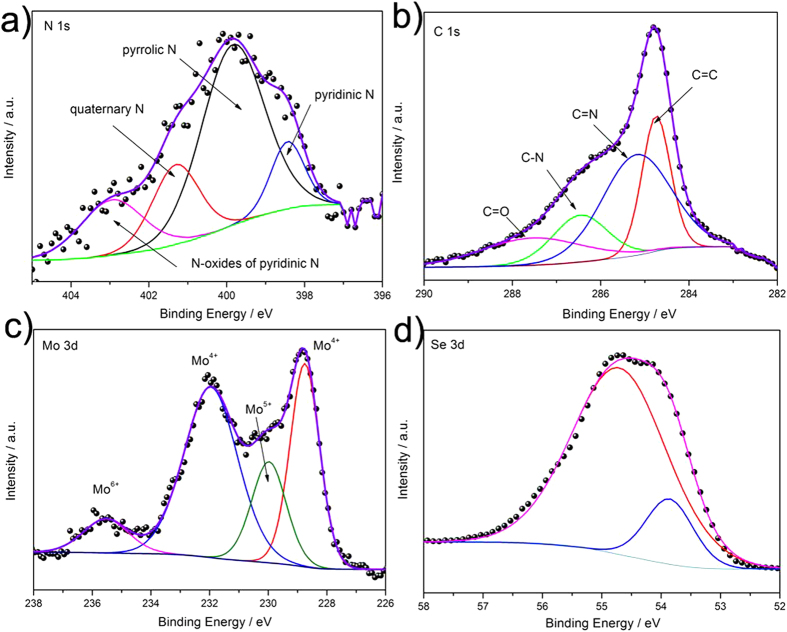
High-resolution XPS spectra of (**a**) the N 1s, (**b**) the C 1s, (**c**) the Mo 3d, and (**d**) the Se 3d regions of HNG-MoSe_2_.

The XPS spectra of HNG-MoSe_2_ and G-MoSe_2_ can further confirm their formations and the N-doping content. The N content increased from ca. 2.5% in G-MoSe_2_ to ca. 12.5% in HNG-MoSe_2_ (Fig. S6). The high-resolution N 1s spectrum of HNG-MoSe_2_ can be fitted to four peaks in Fig. 3a: pyridinic N (398.43 eV), pyrrolic N (399.84 eV), quaternary N (401.30 eV), and N-oxides of pyridinic N (403.11 eV)^33^. Compared to the N 1s spectra of G-MoSe_2_, the HNG-MoSe_2_ spectra possessed more pyridinic (18.64%) and graphitic N (70.43%) than did G-MoSe_2_ (Fig. S7a and Fig. S8), which indicates that the DETA provided pyridinic and graphitic N for the HNG. Previous studies have shown that these two N 1s states are beneficial for reducing redox electrolytes^34^,^35^. These XPS data also suggest that HNG-MoSe_2_, with more pyridinic and graphitic N, could exhibit enhanced electrochemical performance compared to G-MoSe_2_.

In addition, high-resolution C 1s spectra of HNG-MoSe_2_ and G-MoSe_2_ are shown in Fig. 3b and Fig. S9. The intensities of C 1s peaks pertaining to oxygen-containing functional groups decrease dramatically upon solvothermal treatment, revealing a weak-intensity C = O peak at 287.8 eV and new peaks at 285.4 eV (C = N) and 286.5 eV (C–N). Such dramatic changes indicate that the use of DETA to introduce high N-doping into G was efficient for achieving chemical reduction to improve the conductivity of G and further ensure conductivity for electron transfer from the external circuit to the MoSe_2_ catalyst. In addition, the XPS survey spectrum for HNG-MoSe_2_ clearly revealed a Mo:Se stoichiometric ratio of approximately 2:1. The high-resolution XPS of the Mo 3d region of HNG-MoSe_2_ is shown in Fig. 3c. The intense Mo3d_5/2_ (228.8 eV) and Mo3d_3/2_ (232.7 eV) components are characteristic of MoSe_2_. Compared to Mo 3d region of G-MoSe_2_ in the Fig. S7c, the HNG-MoSe_2_ have shown slightly higher content of Mo^4+^ and less Mo^5+^ indicates its higher purity. Se species were determined from the high-resolution XPS spectrum of the Se 3d region (Fig. 3d and Fig. S7d). The main doublet located at binding energies of 53.8 and 54.7 eV corresponds to the Se 3d_5/2_ and Se 3d_3/2_ components, indicating an oxidation state of −2 for Se in MoSe_2_^36^.

## Discussion

**Figure 4:**
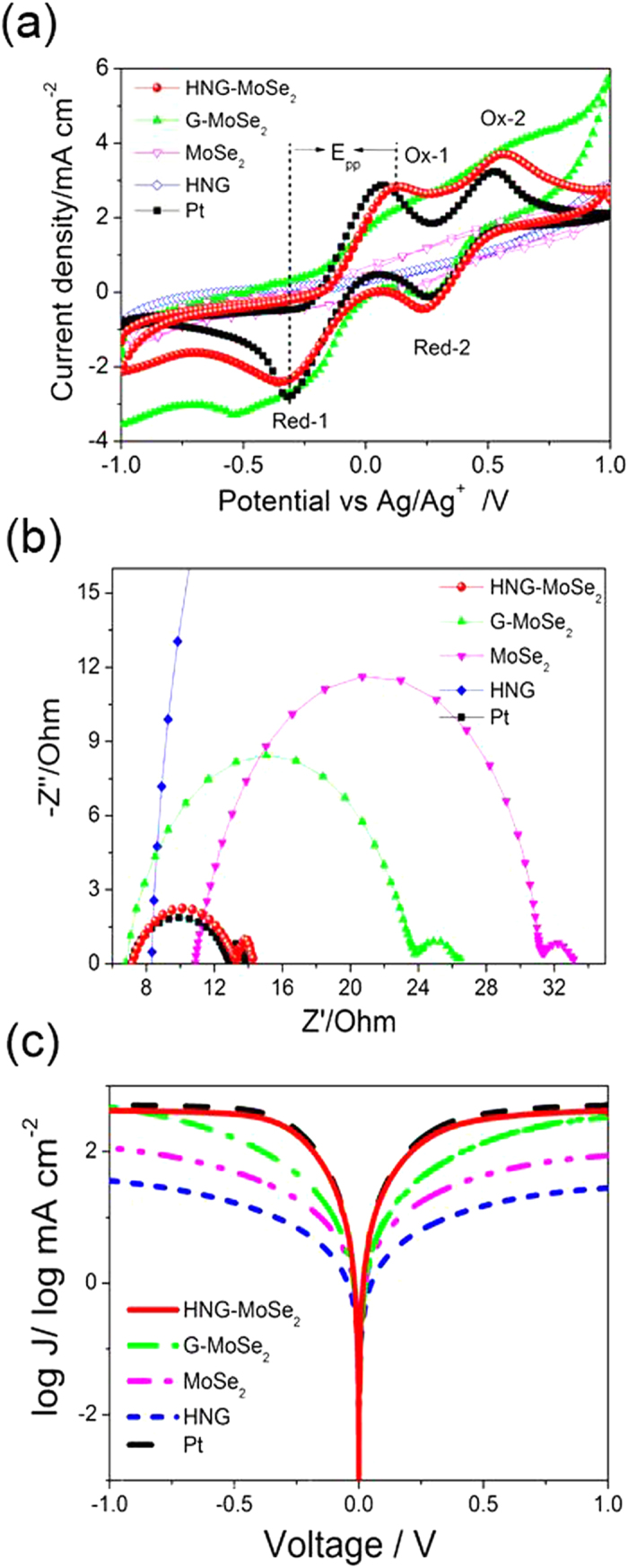
(**a**) Cyclic voltammograms of various CEs in 10  M LiI/1 mM I_2_/acetonitrile solution containing 0.1 M LiClO_4_ as the supporting electrolyte (scan rate: 50 mV s^–1^). (**b**) Nyquist plots at 0 V bias potential and 25 °C obtained for symmetrical cells fabricated using different CEs. The electrolyte for the cells was same as that used for the DSSCs. (**c**) Tafel polarization curves for the CEs used in the EIS experiments.

Prior to evaluating the DSSC devices, the electrocatalytic performance of the CEs was evaluated by means of cyclic voltammetry (CV), electrochemical impedance spectroscopy (EIS), and the Tafel method. Figure 4a shows CV curves for HNG-MoSe_2_, G-MoSe_2_, MoSe_2_, HNG, G, and Pt electrodes. Two characteristic pairs of oxidation and reduction peaks (labeled Ox-1/Red-1 and Ox-2/Red-2 in Fig. 4a) were observed for all the electrodes except G. The Ox-1 and Red-1 peaks are the focus of our analysis, since the CE of a DSSC is responsible for catalyzing the reduction of triiodide (I_3_^–^) to iodide (I^–^). The peak-to-peak separation (*E*_pp_) of the Ox-1/Red-1 pair is inversely correlated with the standard electrochemical catalytic activity for the reduction of I_3_^–^ to I^–^ 37,38. The *E*_pp_ for HNG-MoSe_2_ is 0.44 V (Table 1), which is greater than the *E*_pp_ for Pt (0.36 V). This fact indicates that HNG-MoSe_2_ is a remarkably good electrochemical catalyst for the reduction of I_3_^–^. Moreover, G-MoSe_2_ does not exhibit the characteristic pairs of oxidation and reduction peaks (specifically, no oxidation peak was observed). On the basis of a comprehensive analysis of *E*_pp_ and the current density of the peaks, we deduced that (1) HNG-MoSe_2_ is more conductive than the G-MoSe_2_ due to low O content and more N 1s, which is consistent with the XPS results; and (2) HNG-MoSe_2_, with its above-mentioned unique synergistic effect, is beneficial for catalytic activity compared to G-MoSe_2_. In addition, the HNG-MoSe_2_ exhibit high stability after 200 times CV measurements (Fig. S10), which insure the stability of HNG-MoSe_2_ based device.

The electrocatalytic activity of the CE in DSSCs can further be conveniently evaluated by EIS measurement using a symmetrical dummy cell. Figure 4b shows the Nyquist plots obtained from various dummy cells. According to the Randles-equivalent circuit, the high-frequency intercept on the real axis represents the series resistance (*R*_s_), which is mainly composed of the resistance of the catalyst materials and the resistance of the fluorine-doped SnO_2_ glass substrate. The left semicircle in the middle-frequency range can be attributed to the charge-transfer resistance (*R*_ct_) at the CE/electrolyte interface, which is a pivotal parameter for evaluating the electrocatalytic activities of the CE materials in DSSCs. Low values of *R*_ct_ imply effective reduction of the redox couple at the CE/electrolyte interface. Such reduction favors dye regeneration at the electrolyte/photoanode interface, improving the photocurrent (*J*_sc_). The right semicircle in the low-frequency range can be assigned to the diffusion impedance (Z_N_) of the redox couple (I^–^/I_3_^–^) in the electrolyte^39^,^40^. Clearly, the *R*_s_ value for HNG-MoSe_2_ (7.18 Ω) is comparable to that for Pt (7.14 Ω), indicating that HNG-MoSe_2_ had a good conductive performance. Moreover, the *R*_ct_ value for HNG-MoSe_2_ is 3.04 Ω, which is somewhat close to the *R*_ct_ value for Pt and much lower than the *R*_ct_ values for G-MoSe_2_, MoSe_2_, and G. These data imply that HNG-MoSe_2_ could have excellent catalytic activity for the reduction of triiodide and thus could be used as a less-expensive alternative to Pt as a CE in DSSCs. In addition, Tafel polarization measurements were performed to investigate the catalytic activities of the CEs. A steep slope in the anodic or cathodic branch of the log (current density)–potential (log *J–U*) plot implies a high exchange current density (*J*_0_) on the electrode. Figure 4c shows Tafel curves for dummy cells based on various CEs. Typically, information about *J*_0_ and the limiting diffusion current density (*J*_lim_) can be obtained from the curves at middle potential (with a steep slope) and at high potential, corresponding to the Tafel zone and the diffusion zone, respectively. Both the anodic branch and the cathodic branch showed large slopes for the HNG-MoSe_2_ electrode. These slopes were comparable to those for the Pt electrode and larger than those for the G-MoSe_2_ electrode, suggesting that HNG-MoSe_2_ generated a higher catalytic activity than G-MoSe_2_. These results are consistent with the EIS and CV results. Above all, we consider it is an integral part to generate superior electrochemical performance due to the positive synergistic effect between fullerene-structured MoSe_2_ hollow spheres and highly nitrogen-doped graphene (HNG-MoSe_2_). Beyond that, the XPS results indicate the HNG-MoSe_2_ with more N-doping can be beneficial to the catalyst activity and the high surface-to-volume ratio MoSe_2_ hollow spheres afforded the HNG-MoSe_2_ composite excellent catalytic activity to reduce the redox. Thus we believe both HNG-MoSe_2_ have a positive effect on the reaction with electrolyte because it can act as conductive path to transfer electron from MoSe_2_ or external circuit to electrolyte.

**Figure 5:**
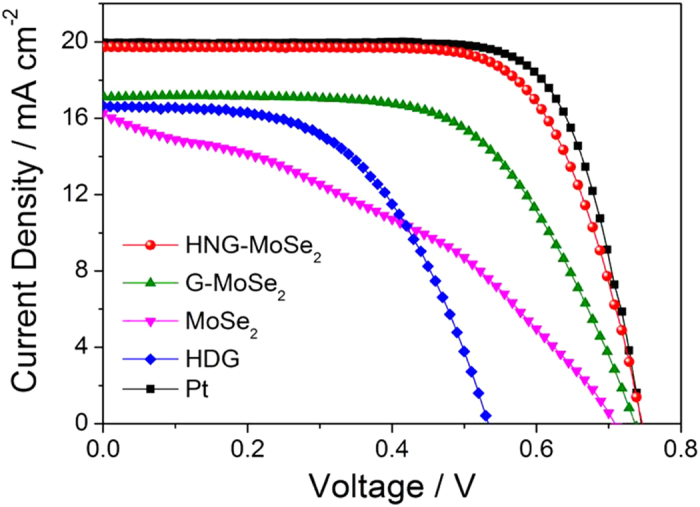
*J–V* curves for DSSCs with HNG-MoSe_2_, G-MoSe_2_, MoSe_2_, HNG, and Pt/FTO (fluorine-doped SnO_2_) CEs measured under AM 1.5 illumination.

The photovoltaic performance of CEs based on HNG-MoSe_2_, G-MoSe_2_, MoSe_2_, HNG, G, and Pt was evaluated. All the DSSC devices adopted the same configuration, using N749 sensitizer dye and I^−^/I_3_^−^ as the redox couple. The DSSCs were irradiated at 100 mW cm^–2^ through a black metal mask with an aperture area of 0.23 cm^2^ 41. The photocurrent density–voltage (*J–V*) curves are shown in Fig. 5; photovoltaic parameters are summarized in Table 1. The photovoltaic parameters of short-circuit current density (*J*_sc_), open-circuit potential (*V*_oc_), and fill factor (FF) for the HNG-MoSe_2_-loaded CEs were 19.73 mA cm^–2^, 724 mV, and 0.70, yielding an overall power conversion efficiency (*η*) of 10.01%. This conversion efficiency is very close to that for a Pt-based device (10.55%) due to the above-mentioned superior synergistic effect between highly N-doped graphene and the unique fullerene-structured MoSe_2_ hollow spheres, which provided an enhanced surface-to-volume ratio. However, when G-MoSe_2_ was implemented as the CE, *η* dropped to 7.34% due to a poor FF (0.60). This low conversion efficiency might have been the result of its poor catalytic activity and conductivity. Therefore, from our experiments, we can conclude that (1) the high FF and *J*_sc_ are ascribable to the decreased *R*_s_ and *R*_ct_; (2) the decreased *R*_s_ benefits from the enhancement of the conductivity, due to the 2D interconnected conductive HNG networks related to MoSe_2_ for efficiently transferring external electrons to MoSe_2_ hollow spheres in the DSSC; (3) the decreased *R*_ct_ benefits from the unique fullerene-structured MoSe_2_ hollow spheres, providing an increased contact area and reduced charge transport length between the redox couple and electrode surface. Finally, compared to other state-of-the-art graphene, inorganic materials based device performance (Table S1), we can conclude that this remarkable improvement highlights the prominent synergistic effect of foreign catalyst and HNG in the hybrid materials.

In summary, fullerene-structured MoSe_2_ hollow spheres anchored on highly N-doped graphene were prepared via a wet chemical process. Structure and composition measurements, including TEM, SEM, and XPS characterizations, confirm that the small molecule DETA enabled the formation of highly N-doped graphene and ultimately yielded HNG-MoSe_2_. The HNG and MoSe_2_ exhibited a superior synergy in which conductivity of the composite material was increased due to the high density N of HNG, while the high surface-to-volume ratio of MoSe_2_ increased the material’s catalytic activity. The resulting DSSC exhibited a conversion efficiency of 10.01%, which is nearly the same as that of a cell with a sputtered Pt counter electrode (10.55%). Besides the DETA used in this study, other small molecules such as ethylenetriamine (ETA) could provide a source of N and serve as a surfactant for creating graphene-based hybrid conductive catalysts. We believe this strategy of fabricating hybrid catalysts with both high conductivity and excellent catalytic activity can be extended to develop high-performance materials for energy storage, catalysis, and optoelectronics.

## Methods

### Preparation of graphene (G)

Graphene oxide (GO),which was prepared from natural graphite by Hummers’ method, was exfoliated by ultrasonication (~120 min) to yield a homogeneous aqueous dispersion of graphene oxide (~5 mg mL^−1^). The graphene (G) was obtained by adding NH_3_•H_2_O (20 mL, ~35 wt%) into 120 ml graphene oxide and then heating at 90 °C for 12 h. The as-obtained graphene (G) hydrogel was dialyzed against deionized water for 24 h and then ultrasonicated for ~2 h to yield a uniform solution of G. After being cooled to room temperature, the hydrogel was dialyzed for 48 h to remove impurities.

### Preparation of HNG-MoSe_2_ and G-MoSe_2_

To synthesize HNG-MoSe_2_, a heterogeneous reaction procedure was developed to deposit fullerene-structured MoSe_2_ hollow spheres on HNG nanosheets. Specifically, 0.3 mmol of H_24_Mo_7_N_6_O_24_•4H_2_O (371 mg) and 10 mmol of Na_2_SeO_3_ were dissolved into a mixed solution of 15 mL of H_2_O and 15 mL of ethylene glycol (EG) and stirred for 30 min to form a homogeneous solution. Diethylenetriamine (DETA, 30 mL) and 30 mLof G (5 mg/mL) were added to the above solution and stirred for 30 min. Afterward, the mixed solution was loaded into a Teflon-lined stainless steel autoclave and heated at 250 °C for 12 h. After being cooled to room temperature, the hydrogel was dialyzed for 24 h to remove impurities. The precipitated products were washed with deionized water and ethyl alcohol three times each, respectively, to remove residual base and then lyophilized for 24 h. The G-MoSe_2_ was prepared under identical conditions, except that DETA was not included in the process.

Next, the as-prepared solution was subjected to ultrasonic treatment for 3 h and spray-coated onto fluorine-doped SnO_2_ (FTO) glass for 10 min. The resulting coated FTO sheets were dried at 300 °C for 3 h in Ar.

### Symmetrical dummy cells for electrochemical catalytic activities

A symmetrical sandwich dummy cell was fabricated from two identical coated FTO sheets, which were separated by 40 μm thick Surlyn (Solaronix, Switzerland) tape, leaving a 1 × 1 cm^2^ active area. The cell was filled with an electrolyte solution through a hole in one FTO support, which was then sealed by a Surlyn seal. The FTO sheet edges were coated by ultrasonic soldering (USS-9200, MBR Electronics) to improve electrical contact.

### Fabrication of dye-sensitized solar cells

Briefly, a photoanode was prepared by screen-printing a dense, transparent TiO_2_ nanoparticle film (~13 μm thickness, 18 nm diameter) and a scattering layer of TiO_2_ nanoparticles (~5 μm thickness, 400 nm diameter) sequentially onto a FTO sheet. The substrate was sintered at 500 °C for 1 h and cooled to 100 °C to generate anatase nanocrystals. After sintering, the TiO_2_ electrode was immersed in TiCl_4_ (40 mM) aqueous solution at 70 °C for 30 min. The film was then annealed at 450 °C for 30 min and sensitized in N749 solution for 24 h. The photoanode and a counter electrode were sealed together with Surlyn film (40 μm thickness) by a hot-press machine. Then, an electrolyte consisting of an acetonitrile solution of 0.6 M (1,2-dimethyl-3-propyl)imidazolium iodide, 0.05 M I_2_, 0.5 M tert-butyl pyridine, and 0.1 M LiI was injected through the hole in the counter electrode of each cell, and the hole was sealed with a Surlyn film and covered with a thin glass slide under heating.

### Characterization and measurement

Morphologies of as-obtained products were observed on a field emission scanning electron microscopic (FE-SEM, FEI Sirion 200). Transmission electron microscopy (TEM, JEOL JEM-2100F) images were obtained under an accelerating voltage of 200 kV. The crystal structures of the counter electrode samples were characterized by powder X-ray diffraction (XRD) using a Goniometer Ultima IV (185 nm) diffractometer with Cu Kα radiation, excited at 40 kV and 40 mA. Raman spectra were taken on a DXR Raman microscope with an excitation length of 532 nm. Elemental composition of composites was analyzed by X-ray photoelectron spectroscopy (XPS) using a Kratos Axis Ultra with monochromatized Al Kα radiation (1486.6 eV).

Electrochemical impedance spectroscopy (EIS) measurements were obtained with an impedance analyzer (Zahner IM6, Germany) at zero bias potential. The impedance studies were carried out under simulated open-circuit conditions in the atmosphere, and with an AC potential amplitude of 5 mV over a frequency range of 0.01–105 Hz in dark conditions. The resultant impedance spectra were analyzed by means of Z-view software. Tafel polarization curves were measured using a Zahner electronchemical workstation at a scan rate of 10 mV s^−1^. Additionally, all the EIS and Tafel measurements were based on a symmetric configurationconsisting of two identical electrodes filled with the same redox electrolyte that was used in the DSSCs. Cyclic voltammetry (CV) was executed in a three-electrode system with different CEs as the working electrode, a platinum wire as the counter electrode, and an Ag/Ag^+^ pseudo reference electrode, which was calibrated with a ferrocene solution after the CV measurements, at a scan rate of 50 mVs^−1^. The electrode was dipped in an anhydrous acetonitrile solution containing 0.1 mM LiClO_4_, 10 mM LiI, and 1 mM I_2_.

For photovoltaic measurements, devices were masked with a thin metal mask to yield an active area of 0.23 cm^2^. *J-V* curves of the DSSCs were measured with a digital source meter (Keithley 2400) under Newport solar simulator giving light with AM 1.5 G spectral distribution, at 100 mW cm^–2^. During *I-V* measurements, a black mask was used with an aperture area of 0.23 cm^2^.

## Additional Information

**How to cite this article**: Bi, E. *et al.* Fullerene-Structured MoSe_2_ Hollow Spheres Anchored on Highly Nitrogen-Doped Graphene as a Conductive Catalyst for Photovoltaic Applications. *Sci. Rep.* **5**, 13214; doi: 10.1038/srep13214 (2015).

## Supplementary Material

Supplementary Information

## Figures and Tables

**Table 1:** Photovoltaic performance of DSSCs using different CEs and corresponding data from CV and EIS spectra[a]

**Device**	***J***_**sc**_ **mA cm**^**–2**^	***V***_**oc**_ **mV**	**FF %**	***η*****%**	***R***_**ct**_ **Ω**	***E***_**pp**_ **V**
HNG-MoSe_2_	19.73	724	70.07	10.01	3.04	0.44
G-MoSe_2_	17.12	710	60.41	7.34	8.49	0.86
MoSe_2_	16.06	704	38.67	4.46	10.24	—
G	16.67	535	54.12	4.83	16.27	—
Pt	19.93	723	73.22	10.55	2.81	0.36

^[a]^*R*_ct_: charge-transfer resistance; *E*_pp_: peak-to-peak voltage separation calculated from CV data.
