# Supplementary material for: Fullerene-Structured MoSe2 Hollow Spheres Anchored on Highly Nitrogen-Doped Graphene as a Conductive Catalyst for Photovoltaic Applications
Source: Sci Rep. 2015 Aug 17;5:13214. doi: 10.1038/srep13214 (PMC4538603; doi:10.1038/srep13214)
Supplement: Supplementary Information [file srep13214-s1.doc]

Supporting Information

for

Fullerene-Structured MoSe_2_ Hollow Spheres Anchored on Highly Nitrogen-Doped Graphene as a Conductive Catalyst for Photovoltaic Applications

Enbing Bi*^a^*, Han Chen*^a^*^*^,Xudong Yang*^a^*, Fei Ye*^a^*, Maoshu Yin*^a^* & Liyuan Han*^a,b*^*

*^a^*State Key Laboratory of Metal Matrix Composites, School of Materials Science and Engineering, Shanghai Jiao Tong University, Shanghai 200240, China

E-mail:[Chen.han@sjtu.edu.cn](mailto:Chen.han@sjtu.edu.cn)

*^b^*Photovoltaic Materials Unit, National Institute for Materials Science, Tsukuba, Ibaraki 305-0047, Japan

E-mail: [HAN.Liyuan@nims.go.jp](mailto:HAN.Liyuan@nims.go.jp)

﻿

**Supplementary Results**


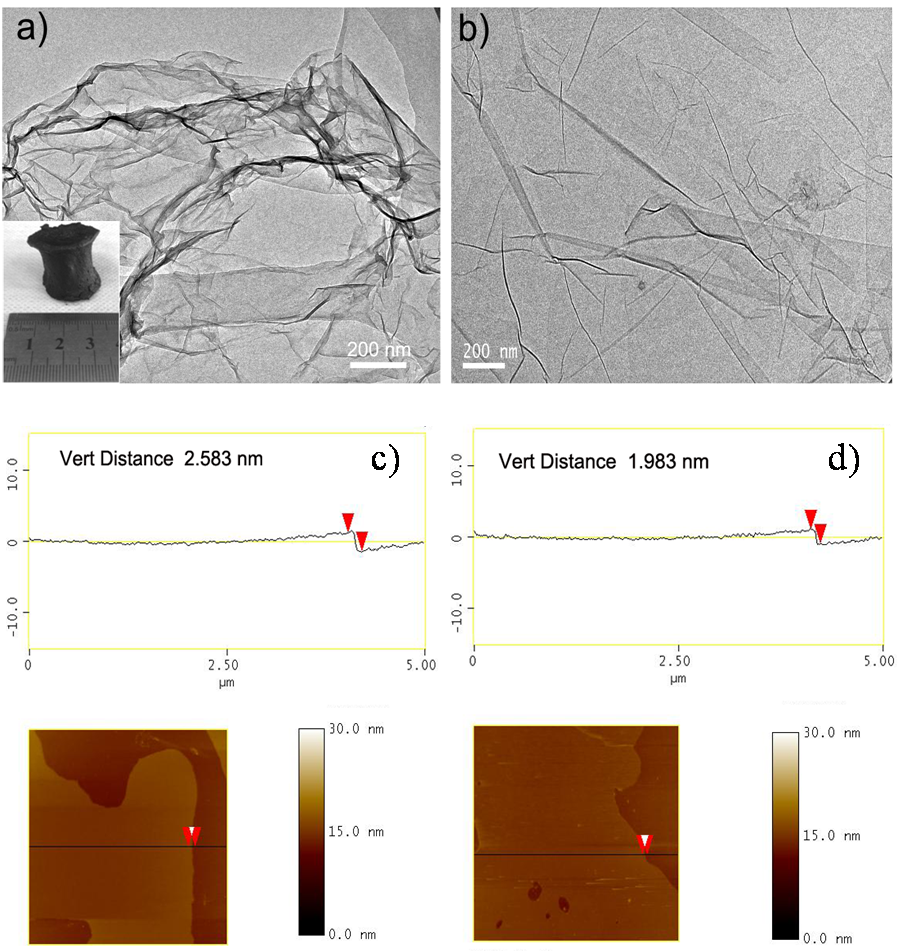


Figure S1. High-resolution transmission electron micrographs of (a) G and (b) GO; atomic force microscope images of (c) G and (d) GO.

G was obtainedby treating GO with heated ammonia (NH_3_·H_2_O), which provided an N-doping source while converting the GO to G with a large surface area for MoSe_2_ growth.


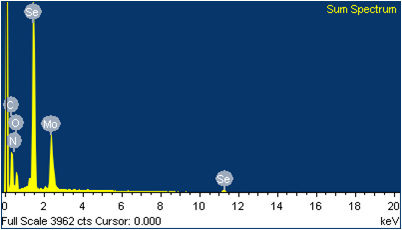


Figure S2. Energy-dispersive X-ray spectroscopy (EDX) of HNG-MoSe_2_.


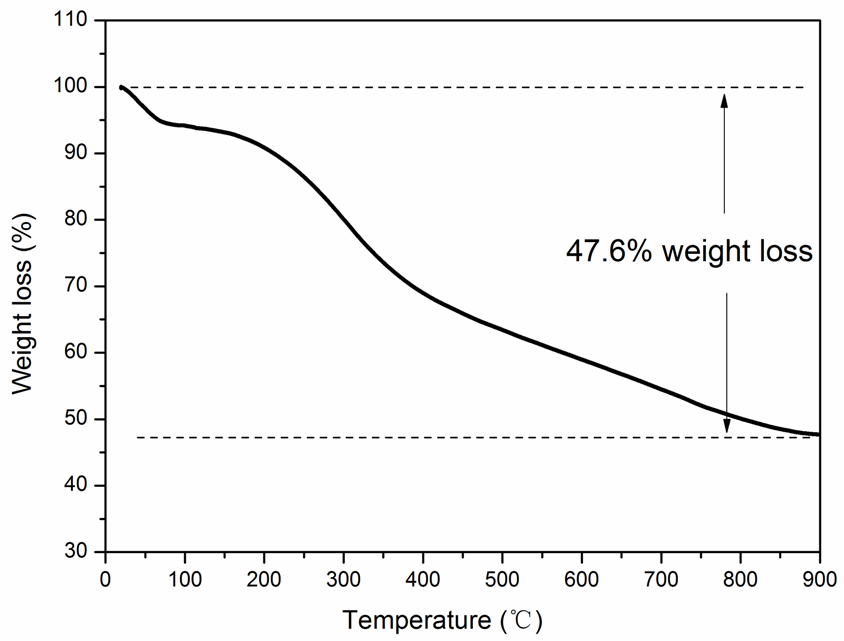


Figure S3. The MoSe_2_ loading amount in the HNG-MoSe_2_ hybrid was about 52.4wt% based on thermogravimetric analysis(TGA).


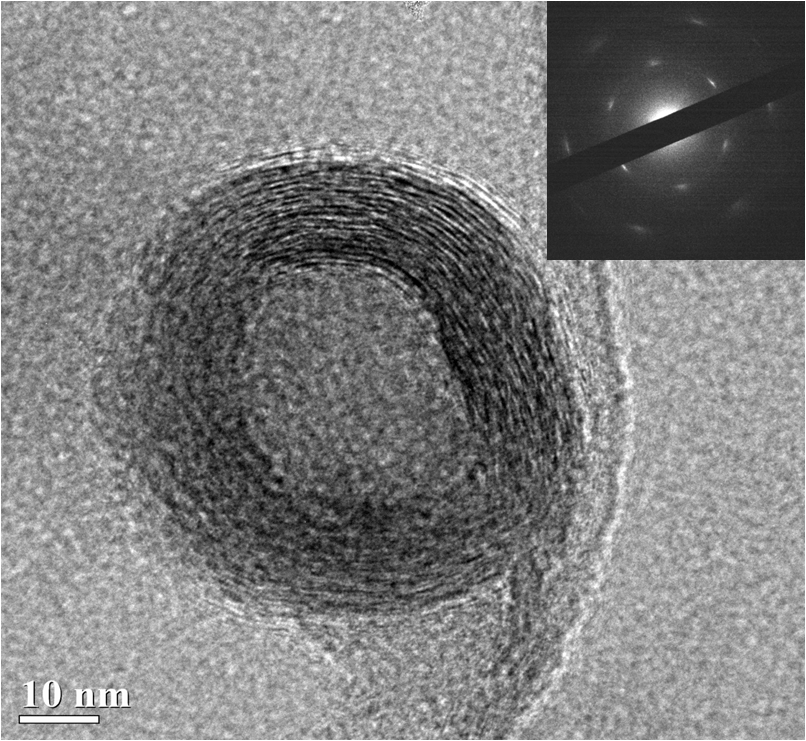


Figure S4. SAED of HNG-MoSe_2_.

**
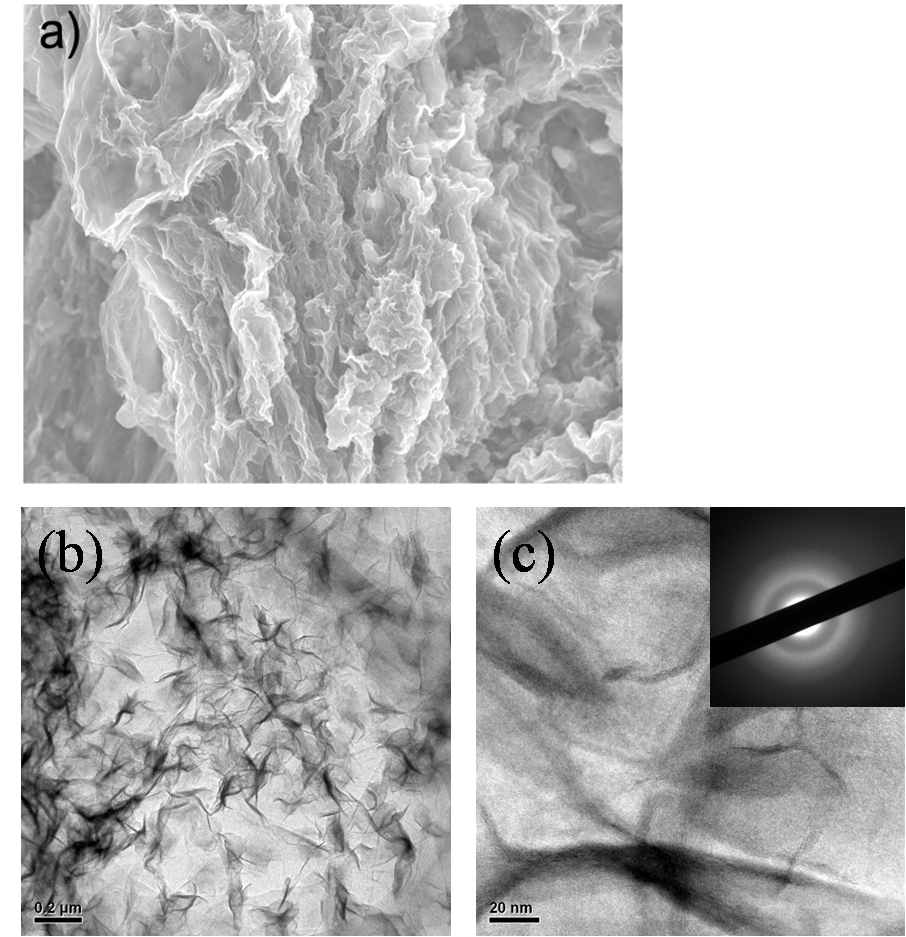
**

Figure S5. (a) SEM image of G-MoSe_2_. (b) TEM image of G-MoSe_2_. (c) SAED image of G-MoSe_2_ with a nanosheet structure on the G layer.

**
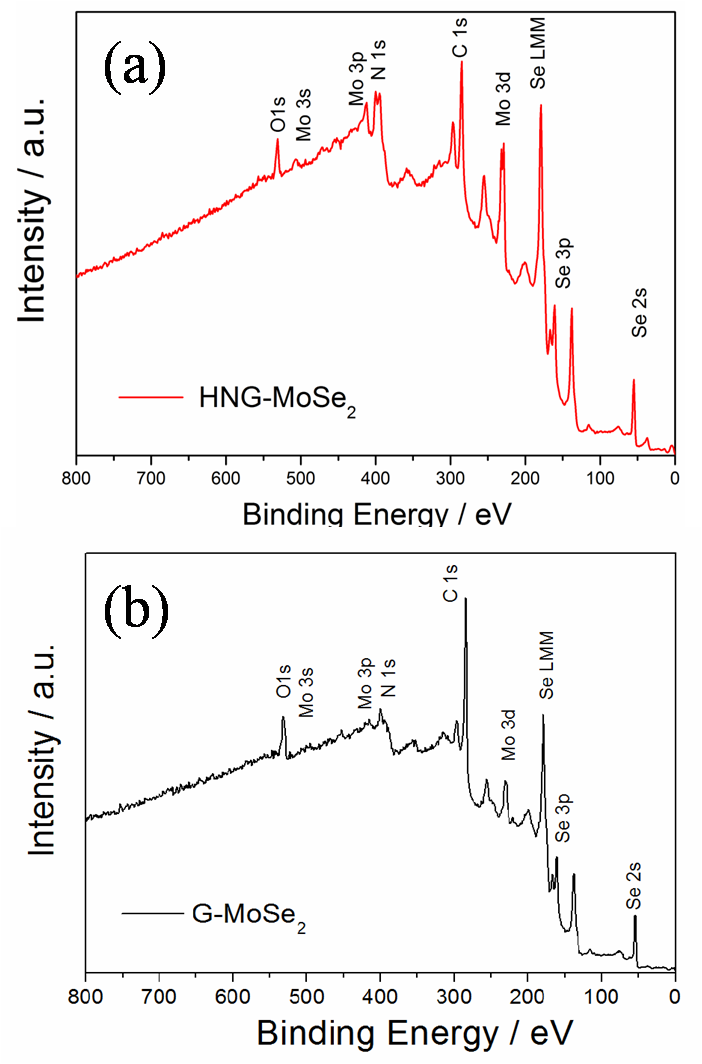
**

Figure S6. XPS spectra of (a) HNG-MoSe_2_ and (b) G-MoSe_2_.

*
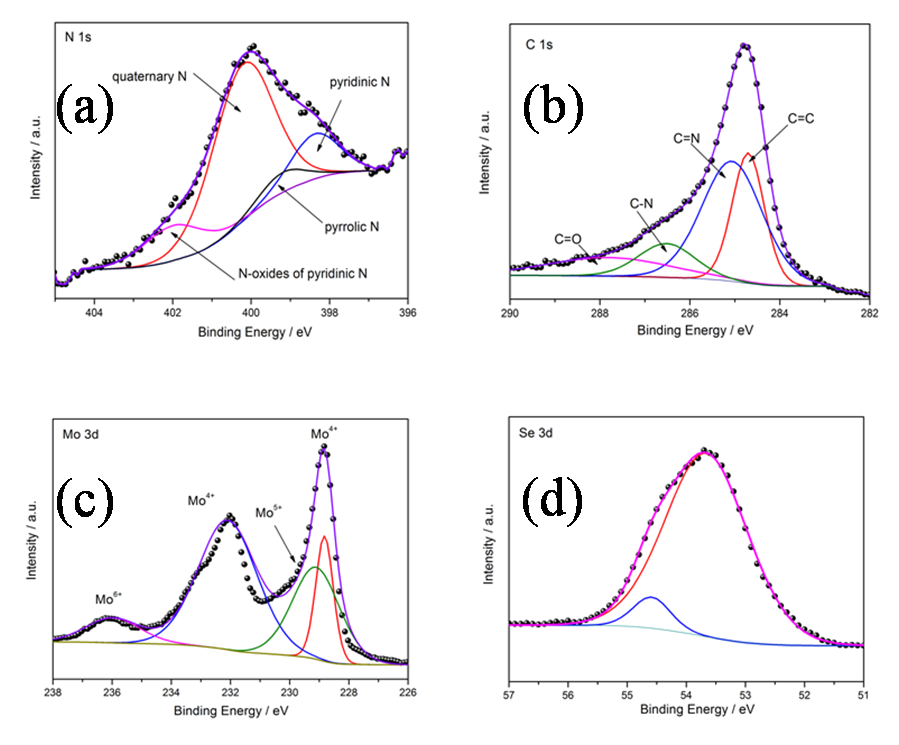
*

Figure S7. XPS spectra of (a) the N1s region for G-MoSe_2_,(b) the C1s region for G-MoSe_2_,(c) the Mo 3d region for G-MoSe_2_, and (d) the Se 3d region for G-MoSe_2_.

**
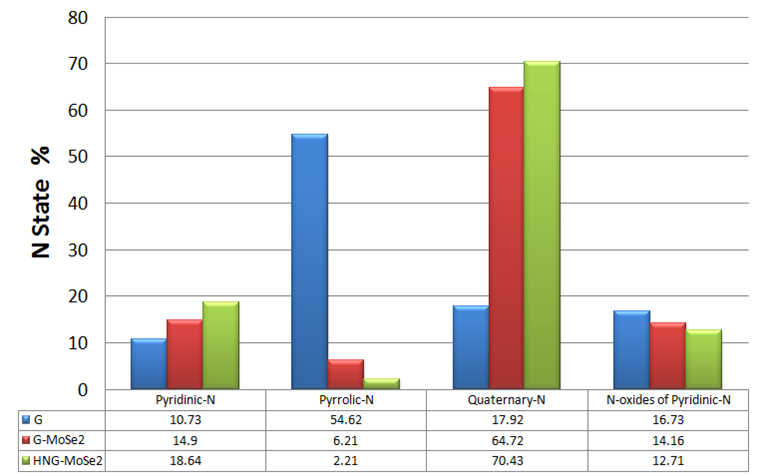
**

Figure S8. The percentages of four types of N1s peaks in HNG-MoSe_2_, G-MoSe_2_, and G.

HNG-MoSe_2_ contained higher percentages of pyridinic and graphitic (quaternary) N (18.64% and 70.43%, respectively) than did G-MoSe_2_ and G. Pyridinic and graphitic N are reported to be more effective than pyrrolic N in improving the electrochemical performance of G. ^1, 2^

1. C. Zhang, L. Fu, N. Liu, M. Liu, Y. Wang and Z. Liu, Adv. Mater., 2011, 23, 1020–1024.
2. L. Lai, J. R. Potts, D. Zhan, L. Wang, C. K. Poh, C. Tang, H. Gong, Z. Shen, J. Lin and R. S. Ruoff, Energy Environ. Sci., 2012, 5, 7936–7942


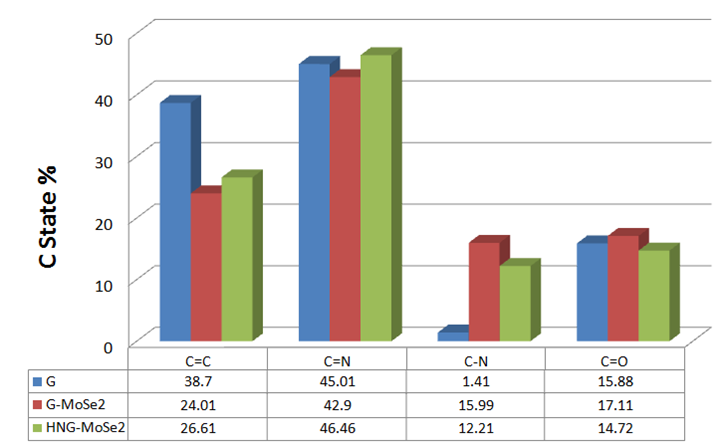


Figure S9.The percentages of four types of C1s peaks in HNG-MoSe_2_, G-MoSe_2_, and G.

The chemical reduction of G can improve the conductivity of graphene-based catalysts. The oxygen in G was mainly bonded with C, such as in C–O, C=O, andO=C–O groups.^3, 4^ Compared to G, the oxygen species C–O, C=O, and O=C–O of the HNG-MoSe_2_ and G-MoSe_2_ FNSwere much less abundant, and the major observedspecies were C=C and C–C, indicating an efficient deoxidization by means of the N-doping process.

3. H. J. Shin, K. K. Kim, A. Benayad, S. M. Yoon, H. K. Park, I. S. Jung, M. H. Jin, H. K. Jeong, J. M. Kim and J. Y. Choi, Adv. Funct. Mater., 2009, 19, 1987–1992.

4. Z. Xu, Y. Zhang, P. Li and C. Gao, ACS Nano, 2012, 6, 7103–7113).


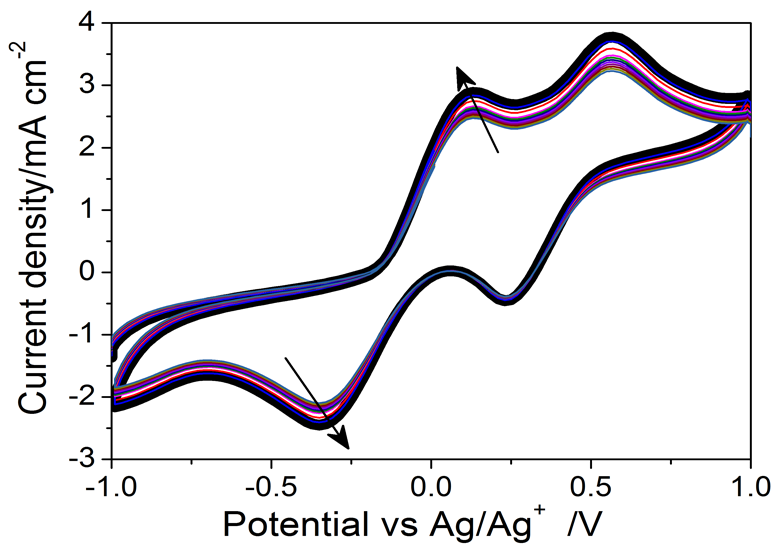


Figure S10.The CV curves of HNG-MoSe_2_ in 200 times scan.

Table S1. A summary table to list the state-of-the-art graphene, inorganic and hybrid materials capable of replacing Pt and the corresponding performances as the counter for DSSCs.

| Types | CEs | *J*_sc_  [mA cm^−2^] | *V*_oc_  [mV] | *FF* | *PCE* [%] | Refs |
| --- | --- | --- | --- | --- | --- | --- |
| TMCs | Co_0.85_Se | 16.98 | 738 | 0.75 | 9.4 | 1 |
|  | WC | 14.17 | 763 | 0.65 | 7.01 | 2 |
|  | NiN | 15.76 | 766 | 0.69 | 8.31 | 3 |
|  | WO_2_ | 14.02 | 808 | 0.64 | 7.25 | 4 |
|  | Co_9_S_8_ | 14.21 | 0.71 | 0.69 | 7.00 | 5 |
| Carbon materials | Graphene | 14.8 | 878 | 0.72 | 9.4 | 6 |
|  | PDDA/GO | 18.77 | 692 | 0.74 | 9.54 | 7 |
|  | GNP（Y123） | 12.7 | 1030 | 0.70 | 9.3 | 8 |
|  | Multiwall CNT | 16.20 | 740 | 0.64 | 7.67 | 9 |
| Hybrids | TiN/MC | 15.3 | 820 | 0.67 | 8.41 | 10 |
|  | RGO/SWCNTs | 12.81 | 860 | 0.76 | 8.37 | 11 |
|  | NiS_2_/RGO | 16.55 | 749 | 0.69 | 8.55 | 12 |
|  | GO/GNP | 15.1 | 885 | 0.67 | 9.3 | 13 |
|  | NDG/CoS | 20.38 | 710 | 0.74 | 10.71 | 14 |
|  | HNG-MoSe_2_ | 19.73 | 724 | 0.70 | 10.01 | This study |

The hybrid electrode (HNG-MoSe_2_) exhibits a conversion efficiency of 10.01%, which is beneficial from the high conductivity and excellent catalytic activity of the HNG-MoSe_2_; the superior synergistic effect between the highly nitrogen-doped graphene and the high surface-to-volume ratio MoSe_2_ hollow spheres afforded the HNG-MoSe_2_ composite these performances.

Compared to the transition metal compounds and carbon material, the highly nitrogen-doped graphene-based materials have more appealing features，including: (1) the Nano-hybrid graphene-based material can endow the electrode a synergistic performance of high conductivity and excellent catalytic activity; (2) though the device based on the graphene counter electrodes can have a relative high performance due to its high conductivity, its low Jsc attribute to the low catalytic performance; (3) this synergistic performance afforded the electrode a decreased *R*_s_ and *R*_ct_, which contribute to the higher Jsc and FF and in return afforded a higher power conversion efficiency of DSSCs.

In addition, the previous core-shell catalyst composed of nitrogen-doped graphene shelled on cobalt sulfide nanocrystals (NDG-CoS) indeed have a good result that the resulting DSSC efficiency based on this hybrid material is 10.71 %, which is comparable to that observed for DSSCs with Pt CEs. However, there are also some problems that we found: (1) the conductivity of these nanocrystals was limited due to the low amount of nitrogen (<5%) in the graphene. Nitrogen incorporation in graphene is expected to improve the catalytic activity of the composites since it enhances the electron-donating ability of the graphene; (2) the CoS nanocrystals is not very stable in the air condition because it apt to form the hydroxide; (3) It is difficult to obtain CoS nanocrystals with high pure phase.

1. Gong, F., Wang, H., Xu, X., Zhou, G. & Wang, Z.-S. In situ growth of Co0.85Se and Ni0. 85Se on conductive substrates as high-performance counter electrodes for dye-sensitized solar cells. Journal of the American Chemical Society 134, 10953-10958 (2012).

2. SukáJang, J. & JináHam, D. Platinum-free tungsten carbides as an efficient counter electrode for dye sensitized solar cells. Chemical communications 46, 8600-8602 (2010).

3. Jiang, Q., Li, G., Liu, S. & Gao, X. Surface-nitrided nickel with bifunctional structure as low-cost counter electrode for dye-sensitized solar cells. The Journal of Physical Chemistry C 114, 13397-13401 (2010).

4. Wu, M., Lin, X., Hagfeldt, A. & Ma, T. A novel catalyst of WO 2 nanorod for the counter electrode of dye-sensitized solar cells. Chemical communications 47, 4535-4537 (2011).

5. Chang, S.-H., Lu, M.-D., Tung, Y.-L. & Tuan, H.-Y. Gram-scale synthesis of catalytic Co9S8 nanocrystal ink as a cathode material for spray-deposited, large-area dye-sensitized solar cells. ACS nano 7, 9443-9451 (2013).

6. Kavan, L., Yum, J.-H. & Grätzel, M. Graphene nanoplatelets outperforming platinum as the electrocatalyst in co-bipyridine-mediated dye-sensitized solar cells. Nano letters 11, 5501-5506 (2011).

7. Xu, X. et al. Electrochemically reduced graphene oxide multilayer films as efficient counter electrode for dye-sensitized solar cells. Scientific reports 3 (2013).

8. Kavan, L., Yum, J.-H., Nazeeruddin, M. K. & Grätzel, M. Graphene nanoplatelet cathode for Co (III)/(II) mediated dye-sensitized solar cells. Acs Nano 5, 9171-9178 (2011).

9. Lee, W. J., Ramasamy, E., Lee, D. Y. & Song, J. S. Efficient dye-sensitized solar cells with catalytic multiwall carbon nanotube counter electrodes. ACS applied materials & interfaces 1, 1145-1149 (2009).

10. Ramasamy, E. et al. Soft-template simple synthesis of ordered mesoporous titanium nitride-carbon nanocomposite for high performance dye-sensitized solar cell counter electrodes. Chemistry of Materials 24, 1575-1582 (2012).

11. Zheng, H., Neo, C. Y. & Ouyang, J. Highly efficient iodide/triiodide dye-sensitized solar cells with gel-coated reduce graphene oxide/single-walled carbon nanotube composites as the counter electrode exhibiting an open-circuit voltage of 0.90 V. ACS applied materials & interfaces 5, 6657-6664 (2013).

12. Li, Z., Gong, F., Zhou, G. & Wang, Z.-S. NiS2/reduced graphene oxide nanocomposites for efficient dye-sensitized solar cells. The Journal of Physical Chemistry C 117, 6561-6566 (2013).

13. Kavan, L., Yum, J.-H. & Graetzel, M. Optically transparent cathode for Co (III/II) mediated dye-sensitized solar cells based on graphene oxide. ACS applied materials & interfaces 4, 6999-7006 (2012).

14. Bi, E. et al. A quasi core–shell nitrogen-doped graphene/cobalt sulfide conductive catalyst for highly efficient dye-sensitized solar cells. Energy & Environmental Science 7, 2637-2641 (2014).
